# On the Spontaneous Evolution of the Fœtus

**Published:** 1828-02

**Authors:** George Jewel


### MIDWIFERY.

### *On the Spontaneous Evolution of the Fœtus.*

By George Jewel, Esq.

When we reflect upon the revolving changes which the several branches of medical science have undergone through succeeding ages, none appear more conspicuous than those which appertain to midwifery. It is, indeed, a fact too palpable to be refuted, that hitherto its principles have neither been so stable or determinate as those which belong to medicine generally: a circumstance, it is fair to presume, attributable to its defective cultivation as a branch of the healing art. Midwifery, unfortunately, has been left, for a long series of years, entirely unprotected by the medical corporate bodies of this kingdom, and, as a natural result, it has never been fully appreciated by students and junior practitioners as an important branch of science. Indeed, it is scarcely exceeding the bounds of truth to say, that very many practitioners of the present day have become acquainted with the mechanical delivery of women in laborious parturition by experience only, and that experience sometimes too dearly bought, while the principles of the science have been left totally uncultivated, frequently unnoticed. If the opinion be correct that the principles of midwifery have been to a certain extent neglected, and it scarcely admits of doubt, it cannot excite surprise that many of the phenomena of parturition should pass disregarded.

The object of this communication is to draw the attention of practitioners to that peculiar condition of the fœtus which obtains in one species of what are termed preternatural labours, and to induce a further development of facts; for it is upon this alone the solid pyramid of science can be raised.

The records of medicine inform us that, among women in all ages, the function of parturition has been not unfrequently interrupted by the cross position of the infant. To convert the preternatural into a natural presentation, or, in other words, to bring the head of the child, when an arm presented, in apposition to the os uteri, must have been (to say the least of it) a very precarious mode of delivery ; in fact, one which seldom could be crowned with success : and, if foiled in the attempt, cutting instruments were had recourse to, under all circumstances, and at any period of the labour, with which they mutilated the child, that it might be extracted piecemeal through the pelvis. In justice, however, it should be stated, that formerly the various conditions and capabilities of the uterine fibre [redacted] not well understood ; and it is upon a knowledge of these that the success of obstetric medicine principally depends.

It had been remarked by some of the most eminent accoucheurs of this and other countries, that in some few cases where an upper extremity of the child was detected as the presenting part soon after the commencement of labour, the nature of the presentation became materially altered before the process was wholly accomplished. Dr. Denman was the first English physician who directed the attention of practitioners to this interesting phenomenon, and which he designated "the spontaneous evolution of the fœtus." The arm or shoulder, he imagined, sometimes presented, but, by the power of the natural efforts, the child turned upon its own axis, the nates ultimately became forced into the pelvic cavity, which compelled the part originally presenting to recede into the uterus. This explanation, however plausible it appeared to some, failed in proving satisfactory to others. Dr. Douglas ably combated the theoretical doctrines of Denman, while at the same time he illustrated his particular views of the subject by the publication of seven cases which had occurred under his own observation.

The two following cases which I have recently met with in my own practice, will prove strong corroborative testimony to the opinion that no spontaneous evolution of the fœtus ever takes place, but that the child, by the extraordinary exertion of the uterine power, is merely expelled in a doubled position.

Case II.—Mary Davey, ætat. thirty-six, residing in Turner's-court, St. Martin's-lane, a poor woman, of short stature, a delicate constitution, and arrived at the full period of utero-gestation, was seized with labour early in the morning of the 1st of June, 1826. On the subsequent day, in the evening, I was sent for by the midwife in attendance. Upon entering the chamber, I observed that the uterine power was exceedingly and most fearfully exerted; large drops of perspiration stood upon her forehead and neck, and there was scarcely a minute's interval between the pains. On making an examination per vaginam, I found the shoulder pressed into the inferior aperture of the pelvis, the ~~head~~ hand having dropped through the os externum. The idea of turning could not be for a moment entertained under existing circumstances, and I found, during a few forcible contractions of the uterus, that the body of the child had become so compressed into the lower division of the pelvis, that a portion of the thorax could be detected almost at the os externum.

Having waited a few minutes to reflect upon the best means I could adopt to meet the exigencies of the case, a pain of augmented severity came on, which caused the woman to scream violently. The midwife now, and as it appeared by design, raised the bedclothes, when I had an opportunity of ascertaining, by ocular demonstration, the precise mechanism of the labour. The arm had already passed through the external parts from the posterior part of the pelvis; by a forcible uterine contraction, the nates appeared, and during the same pain the whole body was expelled. It must be remarked, that the arm neither retracted nor changed its original position in any part of the process. The head was extracted without much difficulty, the child being dead.

The unfortunate subject of this case, whose constitution, as was before remarked, had been always weakly, died the following morning from exhaustion, the effect of the over-active uterine power, and great general disturbance of the system.

Upon an examination of the body, the space in the antero-posterior diameter of the pelvis was found not to exceed three and a half inches, although the child was of the usual size. These are circumstances which materially add to the interest of the case. The uterine parietes were softer than natural, and appeared of a dark gangrenous colour.

Case II.—The wife of a respectable tradesman, residing in the Waterloo-road, forty years of age, and of a robust constitution, was seized with the pains of parturition on Sunday morning, October the 21st, 1827, being the third labour. On my arrival at ten a.m. I found her standing on the floor, notwithstanding the uterus was contracting with extraordinary force every three or four minutes. At my request she immediately got into bed, and having made the usual examination, the right hand of the child was discovered at the os externum, the shoulder under the symphysis pubis. In this, as in the former case, the efforts of the uterus were most powerfully exerted, and I observed that, during the two subsequent pains, a portion of the chest of the fœtus had arrived at the external parts. I now resolved to confide (at least for a time) in the natural powers, and for the following reasons: first, from the parturient action being most excessively severe; secondly, the whole of the shoulder, and part of the thorax, were rapidly approximating to the os externum; and thirdly, the parts were relaxed and lubricated by a plentiful secretion of mucus. That I might not come to any erroneous conclusion regarding the mechanism of the case, I held the hand of the child loosely within my own, by which I was again enabled to perceive that, so far from the original presentation retracting, scarcely any movement whatever took place in it during the process.

Within twenty minutes from the first examination, the breech presented, and the child was expelled, as in the other instance, without artificial assistance.

It is necessary I should remark that the patient had only arrived at the eighth month of pregnancy, and the child was dead; a circumstance attributed to an accident which occurred about ten days previous to the coming on of labour. The mother did well.

In obstetrics, as in the practice of all the other branches of medicine, the interests of science and humanity demand from us a due attention to the capability and resources of nature; that we should investigate into, and be strict observers of, her operations; and that we should unite in drawing a line of demarcation between cases which require the scientific interference of art, and others which may be safely entrusted to the unaided exertion of the natural powers. Dr. Douglas, in his well known pamphlet, published in the year 1819, "the Spontaneous Evolution of the Fœtus," states his conviction that at least one-third "of all cases of cross births" ought not to be subjected to artificial turning. Now, if this belief, founded unquestionably on fact and observation, approaches in any degree to the truth, is it not somewhat extraordinary that so little attention has been paid to a subject which is of such vast importance to the science of obstetrics, and, as a natural consequence, to the best interests of society? After some practical attention to this interesting part of obstetric pathology, I have been led to the following conclusions, not without a hope of seeing them confirmed by the experience of others.

That when the arm and shoulder, together with a portion of the thorax, of the child are detected at the os externum,—

when the uterine vigour is most powerfully exerted, and the woman bears down involuntarily with all her strength,—when the parts are disposed to relax freely, there being a due secretion of mucus, and the mother has previously borne children, the practitioner should not interfere. To these may be superadded a healthy conformation of the pelvis, the full period of gestation not having arrived, and the child being dead. Under all the circumstances above mentioned, more particularly the concurrence of great uterine activity, a fair opportunity should be permitted for the accomplishment of labour by the natural powers alone. I cannot coincide with Dr. Denman's theory, "that a child of the common size, living or but lately dead, in such a state as to possess some degree of resilition, is the best calculated for expulsion in this manner." On the contrary, I am induced to believe that the resilition spoken of would materially contribute to the resistance opposed to the uterine power, and, instead of facilitating, would rather prove an additional obstruction to the accomplishment of the process. That when the arm of the child is protruded into the vagina, it never again recedes to make room for the nates or any other part, as imagined by Dr. Denman, and therefore that the young practitioner would be led into a practical error if he waited for its occurrence as a matter of course.

It is, however, much to be feared that such cases as I have detailed but rarely occur; at least, that they form a small portion only of what are commonly denominated preternatural labours, and, therefore, that, although the practitioner possesses a knowledge of the fact that occasionally, in presentations of the upper extremities, the process of labour is accomplished without the interference of art, yet, as it is not possible to calculate, with any degree of accuracy at the commencement of a case, upon its probable termination, so will it be judicious to turn and deliver as soon as the condition of the uterus will permit; and again, on the other hand, where procrastination has usurped the place of prompt measures,—when the arm is wedged in the pelvis, and the uterus is morbidly exerting its tonic contractions,—and the death of the infant is unquestionably marked,—to pursue a mode of treatment more compatible with the safety of the mother, in removing the arm, or by a further mutilation of the child, to enable the operator to bring it with greater facility through the pelvis.

24, *Sackville-street, Piccadilly; January* 2, 1828.

