# Errata

**Published:** 1828-03

**Authors:** 


ERRATA.—*Page* 103, *line* 22, *for* was *read* were; *page* 104, *line* 14, *for* head *read* hand; *page* 105, *line* 34, *read* on the Spontaneous, &c.

